# Supplementary material for: Validation and Normative Data on the Verbal Fluency Test in a Peruvian Population Ranging from Pediatric to Elderly Individuals
Source: Brain Sci. 2022 Nov 24;12(12):1613. doi: 10.3390/brainsci12121613 (PMC9775499; doi:10.3390/brainsci12121613)
Supplement: Supplementary file 1 [file brainsci-12-01613-s001.zip › brainsci-1909073-supplementary.pdf]

Phonologic Fluency								
**Age Range (years)**	**Educational Level (years)**	**Statistics**	**F**	**A**	**S**	**M**	**R**	**P**
**6 & 8 (n=68)**	**1-6**	**Mean**	4.33	5.39	5.03	5.45	4.64	6.42
		**SEM**	0.293	0.329	0.319	0.342	0.287	0.301
		**SD**	2.402	2.691	2.611	2.803	2.353	2.463
**9 & 11 (n=77)**	**1-6**	**Mean**	6.39	7.45	6.92	7.68	7.42	8.58
		**SEM**	0.345	0.373	0.33	0.323	0.315	0.399
		**SD**	3.027	3.275	2.896	2.83	2.764	3.499
**12 & 14 (n=98)**	**1-6**	**Mean**	10.11	10.79	9.9	10.79	10.39	12.33
		**SEM**	0.377	0.369	0.366	0.387	0.4	0.388
		**SD**	3.733	3.656	3.625	3.835	3.96	3.842
**15 & 17 (n=369)**	**7-11**	**Mean**	10.34	11.03	10.6	11.64	11.02	12.68
		**SEM**	0.194	0.183	0.173	0.182	0.18	0.211
		**SD**	3.721	3.511	3.325	3.49	3.464	4.051
**18 & 20 (n=626)**	**7-11**	**Mean**	9.68	9.89	9.9	10.9	10.48	12.05
		**SEM**	0.152	0.146	0.143	0.143	0.144	0.16
		**SD**	3.791	3.652	3.571	3.568	3.593	3.993
**21 & 25 (n=378)**	**7-11**	**Mean**	10.55	10.45	10.71	11.37	11.02	12.4
		**SEM**	0.337	0.186	0.195	0.197	0.194	0.202
		**SD**	6.55	3.616	3.799	3.824	3.778	3.921
**26 & 30 (n=63)**	**7-11**	**Mean**	10.48	10.57	10.6	11.56	11.1	12.03
		**SEM**	0.497	0.475	0.457	0.48	0.466	0.498
		**SD**	3.947	3.77	3.63	3.813	3.697	3.951
**31 & 40 (n=29)**	**7-11**	**Mean**	11.03	10.9	11.69	11.59	12.59	13
		**SEM**	0.681	0.521	0.45	0.597	0.603	0.559
		**SD**	3.669	2.807	2.422	3.213	3.246	3.012

Supplementary Table 1. Total scores for phonologic fluency, distributed by age and educational years, until 41 years old.

Phonologyc fluency								
**Age Range (years)**	**Educational Level (years)**	**Statistics**	**F**	**A**	**S**	**M**	**R**	**P**
**41 & 50 (n=13)**	**1 – 6**	**Mean**	4.08	6.08	4.08	4.08	4	6.08
		**SEM**	0.746	0.746	0.851	0.851	0.809	0.851
		**SD**	2.691	2.691	3.068	3.068	2.915	3.068
**41 & 50 (n=26)**	**7 - 11**	**Mean**	8.27	9.92	8.46	8.19	8.65	10.15
		**SEM**	1.088	1	1.117	1.032	1.183	1.034
		**SD**	5.547	5.098	5.694	5.261	6.033	5.274
**41 & 50 (n=25)**	**12 <**	**Mean**	11.48	13.48	11.4	11.4	11.48	13.4
		**SEM**	1.041	1.041	1.047	1.036	1.033	1.047
		**SD**	5.205	5.205	5.236	5.18	5.165	5.236
**51 & 55 (n=27)**	**1 – 6**	**Mean**	8.59	10.59	8.74	8.67	8.59	10.74
		**SEM**	0.917	0.917	0.916	0.898	0.911	0.916
		**SD**	4.766	4.766	4.76	4.666	4.733	4.76
**51 & 55 (n=77)**	**7 - 11**	**Mean**	7.62	9.58	7.75	7.71	7.7	9.82
		**SEM**	0.492	0.49	0.501	0.512	0.514	0.509
		**SD**	4.32	4.302	4.395	4.495	4.51	4.465
**51 & 55 (n=64)**	**12 <**	**Mean**	11.2	13.2	11.2	11.2	11.02	13.2
		**SEM**	0.537	0.537	0.55	0.548	0.523	0.55
		**SD**	4.299	4.299	4.398	4.387	4.18	4.398
**55 & 60 (n=65)**	**1 – 6**	**Mean**	6.77	8.77	6.68	6.66	6.78	8.68
		**SEM**	0.563	0.563	0.537	0.548	0.551	0.537
		**SD**	4.537	4.537	4.327	4.42	4.446	4.327
**55 & 60 (n=64)**	**7 - 11**	**Mean**	7.88	9.88	7.94	7.91	7.86	9.94
		**SEM**	0.541	0.541	0.527	0.54	0.54	0.527
		**SD**	4.33	4.33	4.216	4.319	4.324	4.216
**55 & 60 (n=58)**	**12 <**	**Mean**	9.55	11.55	9.59	9.66	9.84	11.59
		**SEM**	0.592	0.592	0.612	0.6	0.58	0.612
		**SD**	4.512	4.512	4.664	4.567	4.416	4.664
**61 & 65 (n=55)**	**1 – 6**	**Mean**	6.33	8.33	6.31	6.35	6.33	8.31
		**SEM**	0.567	0.567	0.573	0.551	0.567	0.573
		**SD**	3.971	3.971	4.011	3.854	3.971	4.011
**61 & 65 (n=60)**	**7 - 11**	**Mean**	6.38	8.38	6.3	6.27	6.35	8.3
		**SEM**	0.499	0.499	0.508	0.509	0.493	0.508
		**SD**	3.862	3.862	3.933	3.94	3.817	3.933
**61 & 65 (n=34)**	**12 <**	**Mean**	10.82	12.82	10.91	11.03	11.18	12.91
		**SEM**	0.808	0.808	0.814	0.824	0.833	0.814
		**SD**	4.713	4.713	4.744	4.802	4.858	4.744

Supplementary Table 2. Total scores for phonologic fluency, distributed by age and educational years, until 66 years old.

Phonologyc fluency								
Age Range (years)	Educational Level (years)	Statistics	F	A	S	M	R	P
**66 & 70 (n=69)**	**1 – 6**	Mean	4.67	6.67	4.76	4.82	4.7	6.76
		SEM	0.401	0.401	0.402	0.415	0.416	0.402
		SD	3.26	3.26	3.263	3.374	3.383	3.263
**66 & 70 (n=57)**	**7 – 11**	Mean	7.19	9.19	7.33	7.26	7.33	9.33
		SEM	0.558	0.558	0.555	0.564	0.556	0.555
		SD	4.211	4.211	4.189	4.262	4.198	4.189
**66 & 70 (n=22)**	**12 <**	Mean	10.5	12.5	10.36	10.82	10.55	12.36
		SEM	0.845	0.845	0.81	0.864	0.87	0.81
		SD	3.961	3.961	3.799	4.055	4.079	3.799
**71 & 75 (n=32)**	**1 – 6**	Mean	5.64	7.64	5.75	5.75	5.75	7.75
		SEM	0.63	0.63	0.603	0.581	0.599	0.603
		SD	3.336	3.336	3.193	3.075	3.17	3.193
**71 & 75 (n=24)**	**7 – 11**	Mean	6.13	8.13	6.17	6.17	6.08	8.17
		SEM	0.559	0.559	0.541	0.491	0.521	0.541
		SD	2.74	2.74	2.648	2.408	2.552	2.648
**71 & 75 (n=14)**	**12 <**	Mean	10	12	10.43	10.21	10.21	12.43
		SEM	1.351	1.351	1.481	1.351	1.438	1.481
		SD	5.054	5.054	5.543	5.056	5.381	5.543
**76 & 80 (n=37)**	**1 – 6**	Mean	5.42	7.42	5.5	5.58	5.64	7.5
		SEM	0.601	0.601	0.6	0.601	0.571	0.6
		SD	3.605	3.605	3.598	3.605	3.424	3.598
**76 & 80 (n=13)**	**7 – 11**	Mean	6.54	8.54	6.85	6.69	6.31	8.85
		SEM	1.475	1.475	1.552	1.384	1.42	1.552
		SD	5.317	5.317	5.595	4.99	5.122	5.595
**76 & 80 (n=05)**	**12 <**	Mean	7.6	9.6	7.6	7.8	7.6	9.6
		SEM	0.98	0.98	0.98	0.917	0.812	0.98
		SD	2.191	2.191	2.191	2.049	1.817	2.191
**81 & 85 (n=31)**	**1 – 6**	Mean	6.1	8.1	6.07	6.03	6	8.07
		SEM	0.684	0.684	0.669	0.649	0.695	0.669
		SD	3.745	3.745	3.667	3.557	3.806	3.667
**81 & 85 (n=06)**	**7 – 11**	Mean	5.25	7.25	5.25	5.5	5	7.25
		SEM	0.75	0.75	0.75	0.866	0.577	0.75
		SD	1.5	1.5	1.5	1.732	1.155	1.5
**86 < (n=16)**	**1 – 6**	Mean	5.23	7.23	5.31	5.54	5.46	7.31
		SEM	1.549	1.549	1.491	1.567	1.608	1.491
		SD	5.585	5.585	5.376	5.651	5.797	5.376

Supplementary Table 3. Total scores for phonologic fluency, distributed by age and educational years, after 65 years old.

Age Range (years)	Educational Level (years)	Statistics	Animals	Fruits
41 & 50 (n=13)	1 - 6	Mean	11.31	11.62
		SEM	1.375	1.243
		SD	4.956	4.482
41 & 50 (n=26)	7 – 11	Mean	14.88	14.81
		SEM	1.169	1.228
		SD	5.962	6.261
41 & 50 (n=25)	12 <	Mean	15.56	15.52
		SEM	1.506	1.398
		SD	7.528	6.989
51 & 55 (n=27)	1 - 6	Mean	15.37	15.3
		SEM	1.15	1.175
		SD	5.975	6.107
51 & 55 (n=77)	7 – 11	Mean	13.43	13.52
		SEM	0.578	0.586
		SD	5.072	5.144
51 & 55 (n=64)	12 <	Mean	14.81	15.06
		SEM	0.722	0.757
		SD	5.779	6.052
55 & 60 (n=65)	1 - 6	Mean	13.17	13.2
		SEM	0.63	0.619
		SD	5.082	4.994
55 & 60 (n=64)	7 – 11	Mean	14.16	14.14
		SEM	0.734	0.715
		SD	5.87	5.718
55 & 60 (n=58)	12 <	Mean	15.83	16
		SEM	0.725	0.744
		SD	5.519	5.663
61 & 65 (n=55)	1 - 6	Mean	12.37	12.53
		SEM	0.789	0.774
		SD	5.522	5.42
61 & 65 (n=60)	7 – 11	Mean	12.2	12.22
		SEM	0.665	0.717
		SD	5.155	5.554
61 & 65 (n=34)	12 <	Mean	17.26	17.38
		SEM	0.98	0.98
		SD	5.717	5.716

Supplementary Table 4. Total scores for semantic fluency, distributed by age and educational years, until 66 years old.

Semantic Fluency				
**Age Range (years)**	Educational Level (years)	Statistics	Animals	Fruits
**66 & 70 (n=69)**	**1 – 6**	Mean	11.2	11.18
		SEM	0.703	0.707
		SD	5.714	5.743
**66 & 70 (n=57)**	**7 – 11**	Mean	13.35	13.4
		SEM	0.79	0.774
		SD	5.963	5.843
**66 & 70 (n=22)**	**12 <**	Mean	16.59	16.55
		SEM	1.023	1.048
		SD	4.797	4.916
**71 & 75 (n=32)**	**1 – 6**	Mean	11.61	11.5
		SEM	1.051	1.047
		SD	5.56	5.541
**71 & 75 (n=24)**	**7 – 11**	Mean	11.88	12.04
		SEM	1.138	1.116
		SD	5.574	5.465
**71 & 75 (n=14)**	**12 <**	Mean	13.36	13.29
		SEM	1.377	1.424
		SD	5.153	5.327
**76 & 80 (n=37)**	**1 – 6**	Mean	11.69	11.5
		SEM	1.002	1.024
		SD	6.013	6.144
**76 & 80 (n=13)**	**7 – 11**	Mean	13.23	13.69
		SEM	1.246	1.351
		SD	4.494	4.871
**76 & 80 (n=05)**	**12 <**	Mean	18.2	17.6
		SEM	1.562	1.568
		SD	3.493	3.507
**81 & 85 (n=31)**	**1 – 6**	Mean	11.33	11.27
		SEM	0.991	0.996
		SD	5.429	5.458
**81 & 85 (n=06)**	**7 – 11**	Mean	15.25	16
		SEM	2.097	2.415
		SD	4.193	4.83
**86 < (n=16)**	**1 – 6**	Mean	6.5	6.5
		SEM	2.5	2.5
		SD	3.536	3.536
**66 & 70 (n=69)**	**1 – 6**	Mean	11.23	11.31
		SEM	1.387	1.416
		SD	5.003	5.105

Supplementary Table 5. Total scores for semantic fluency, distributed by age and educational years, after 65 years old.

Age Range (years)	Percentiles	F	A	S	M	R	P	Animals	Fruits
06 – 08 Females	5	1.00	1.00	1.00	1.00	1.00	3.00	6.50	5.00
	10	2.00	2.00	2.00	2.00	1.00	4.00	7.00	6.00
	15	2.00	2.50	2.00	2.00	2.50	4.50	8.00	6.50
	25	3.00	3.00	3.00	3.50	4.00	6.00	10.00	7.00
	50	4.00	5.00	4.00	6.00	5.00	8.00	12.00	8.00
	75	5.00	7.00	6.00	8.00	7.00	9.00	14.00	11.00
	85	6.00	7.00	7.00	8.50	7.00	9.00	16.00	11.00
	90	6.00	9.00	8.00	11.00	7.00	10.00	16.00	12.00
	95	11.00	13.00	9.50	12.00	9.00	12.00	19.50	13.50

Supplementary Table 6. Percentiles Box by Age Range (between 06 and 08 years old), and by Educational level (Between 1 – 6 years) for females.

**Age Range (years)**	**Percentiles**	**F**	**A**	**S**	**M**	**R**	**P**	**Animals**	**Fruits**
**06 – 08 Males**	5	.00	.95	1.00	1.00	.95	1.95	6.00	3.95
	10	1.90	2.00	2.00	2.90	1.00	2.00	7.90	4.00
	15	2.00	3.00	2.00	3.00	2.00	3.00	8.00	5.00
	25	2.75	4.00	3.00	3.00	2.00	4.75	10.75	5.75
	50	4.00	5.00	5.50	4.00	4.00	6.00	13.00	7.50
	75	6.00	8.00	7.25	7.00	6.00	7.25	15.00	9.00
	85	7.15	8.00	9.15	8.00	7.00	8.00	17.15	10.15
	90	8.00	9.00	10.00	9.10	7.00	8.10	19.20	11.00
	95	8.15	10.10	10.00	10.10	9.10	9.15	22.00	16.10

Supplementary Table 7. Percentiles Box by Age Range (between 06 and 08 years old), and by Educational level (Between 1 – 6 years) for males.

**Age Range (years)**	**Percentiles**	**F**	**A**	**S**	**M**	**R**	**P**	**Animals**	**Fruits**
**9-11**	5	2.80	3.80	2.80	3.80	2.80	2.80	9.40	7.00
**Females**	10	3.00	4.00	3.60	4.60	3.00	4.60	11.00	7.60
	15	3.00	5.00	4.00	5.00	5.00	6.00	11.40	8.00
	25	4.00	6.00	5.00	6.00	5.00	6.00	12.00	9.00
	50	6.00	7.00	7.00	7.00	8.00	8.00	15.00	10.00
	75	8.00	9.00	9.00	10.00	9.00	11.00	18.00	12.00
	85	10.00	11.00	10.00	11.60	10.00	13.00	19.60	13.00
	90	11.40	12.20	10.00	13.00	11.00	14.00	20.00	14.40
	95	13.40	14.20	12.20	14.20	11.40	15.40	21.20	15.40

Supplementary Table 8. Percentiles Box by Age Range (between 9 and 11 years old), and by Educational level (Between 1 – 6 years) for females.

Age Range (years)	Percentiles	F	A	S	M	R	P	Animals	Fruits
**9-11**	5	2.00	1.00	2.15	3.00	3.15	2.15	8.15	5.00
**Males**	10	3.00	2.30	3.00	3.30	4.00	4.00	10.00	6.00
	15	3.00	3.45	3.45	5.00	4.45	4.45	10.90	7.00
	25	4.00	4.75	5.00	6.00	5.00	5.75	13.00	8.00
	50	6.00	7.00	7.00	7.50	7.00	8.00	16.00	10.50
	75	7.25	9.25	8.25	9.00	9.00	11.25	19.25	13.00
	85	10.55	11.55	9.55	10.00	10.55	12.55	20.00	13.00
	90	11.00	13.00	11.70	11.70	11.70	13.00	20.00	14.00
	95	12.85	13.85	13.85	13.00	13.70	14.85	21.00	16.00

Supplementary Table 9. Percentiles Box by Age Range (between 9 and 11 years old), and by Educational level (Between 1 – 6 years) for males.

Age Range (years)	Percentiles	F	A	S	M	R	P	Animals	Fruits
**41-50**	5	1.00	3.00	1.00	1.00	1.00	3.00	5.00	6.00
**Females**	10	1.00	3.00	1.00	1.00	1.00	3.00	5.00	6.00
	15	1.20	3.20	1.00	1.20	1.00	3.00	5.00	6.20
	25	2.00	4.00	1.00	2.00	1.00	3.00	5.00	7.00
	50	4.00	6.00	4.00	4.00	4.00	6.00	14.00	14.00
	75	6.00	8.00	7.00	6.00	6.00	9.00	15.00	15.00
	85	8.40	10.40	9.40	9.20	8.40	11.40	21.40	19.80
	90	.	.	.	.	.	.	.	.
	95	.	.	.	.	.	.	.	.

Supplementary Table 10. Percentiles Box by Age Range (between 41 and 50 years old), and by Educational level (Between 1 – 6 years) for females.

Age Range (years)	Percentiles	F	A	S	M	R	P P	Animals	Fruits
**41-50**	5	2.00	4.00	1.00	1.00	1.00	3.00	7.00	6.00
**Males**	10	2.00	4.00	1.00	1.00	1.00	3.00	7.00	6.00
	15	2.00	4.00	1.05	1.00	1.05	3.05	7.00	6.05
	25	2.00	4.00	1.75	1.00	1.75	3.75	7.00	6.75
	50	3.00	5.00	3.00	3.00	3.00	5.00	10.00	10.50
	75	6.75	8.75	6.75	7.50	7.50	8.75	12.50	12.75
	85	8.85	10.85	8.85	8.90	8.90	10.85	13.90	14.85
	90	.	.	.	.	.	.	.	.
	95	.	.	.	.	.	.	.	.

Supplementary Table 11. Percentiles Box by Age Range (between 41 and 50 years old), and by Educational level (Between 1 – 6 years) for males.

Age Range (years)	Percentiles	F	A	S	M	R	P	Animals	Fruits
**51-55**	5	.00	2.00	-1.00	-1.00	-1.00	1.00	2.00	1.00
**Females**	10	.80	2.80	1.40	1.40	.60	3.40	7.60	7.40
	15	3.10	5.10	3.40	3.40	3.10	5.40	9.00	9.00
	25	4.00	6.00	4.00	4.00	4.00	6.00	9.00	9.00
	50	7.00	9.00	7.00	7.00	6.00	9.00	14.00	15.00
	75	14.50	16.50	14.00	14.50	14.00	16.00	22.00	21.00
	85	17.00	19.00	16.00	16.30	16.00	18.00	22.00	22.30
	90	17.00	19.00	16.20	17.00	16.20	18.20	22.20	23.00
	95	.	.	.	.	.	.	.	.

Supplementary Table 12. Percentiles Box by Age Range (between 51 and 55 years old), and by Educational level (Between 1 – 6 years) for females.

Age Range (years)	Percentiles	F	A	S	M	R	P	Animals	Fruits
**51-55**	5	4.00	6.00	4.00	4.00	4.00	6.00	7.00	5.00
**Males**	10	4.20	6.20	4.20	4.20	4.20	6.20	7.20	5.40
	15	5.30	7.30	5.30	5.30	5.30	7.30	8.30	7.60
	25	6.00	8.00	6.00	6.75	6.75	8.00	10.50	11.25
	50	9.00	11.00	10.00	9.00	10.00	12.00	17.50	17.00
	75	10.50	12.50	11.00	10.50	10.75	13.00	20.75	20.50
	85	12.35	14.35	14.00	12.35	13.00	16.00	23.00	22.35
	90	12.90	14.90	14.00	12.90	13.00	16.00	23.00	22.90
	95	.	.	.	.	.	.	.	.

Supplementary Table 13. Percentiles Box by Age Range (between 51 and 55 years old), and by Educational level (Between 1 – 6 years) for males.

Age Range (years)	Percentiles	F	A	S	M	R	P	Animals	Fruits
**56-60**	5	1.00	3.00	1.00	1.00	1.00	3.00	5.00	5.00
**Females**	10	1.00	3.00	1.50	1.00	1.00	3.50	5.50	6.00
	15	1.00	3.00	2.00	1.00	1.75	4.00	6.75	7.00
	25	4.00	6.00	4.00	4.00	4.00	6.00	9.00	9.00
	50	7.00	9.00	6.00	7.00	7.00	8.00	13.00	13.00
	75	10.00	12.00	10.00	10.00	10.00	12.00	19.00	17.75
	85	13.25	15.25	13.00	13.00	13.25	15.00	19.25	19.25
	90	14.00	16.00	14.00	13.50	14.00	16.00	20.00	20.00

	95	14.00	16.00	14.00	14.00	14.00	16.00	22.00	22.75

Supplementary Table 14. Percentiles Box by Age Range (between 56 and 60 years old), and by Educational level (Between 1 – 6 years) for females.

**Age Range (years)**	**Percentiles**	**F**	**A**	**S**	**M**	**R**	**P**	**Animals**	**Fruits**
**56-60**	5	1.00	3.00	1.00	1.00	1.00	3.00	6.30	5.40
**Males**	10	1.00	3.00	1.00	1.00	1.20	3.00	9.00	9.00
	15	1.30	3.30	1.30	1.00	2.00	3.30	9.60	9.60
	25	2.00	4.00	2.00	2.00	2.00	4.00	11.00	11.00
	50	6.00	8.00	6.00	6.00	6.00	8.00	11.00	12.00
	75	8.00	10.00	8.00	8.00	8.00	10.00	14.50	14.50
	85	10.00	12.00	9.00	9.00	10.00	11.00	20.40	20.70
	90	13.20	15.20	12.20	13.00	13.20	14.20	21.80	21.80
	95	16.70	18.70	15.70	15.80	15.80	17.70	22.00	22.00

Supplementary Table 15. Percentiles Box by Age Range (between 56 and 60 years old), and by Educational level (Between 1 – 6 years) for males.

**Age Range (years)**	**Percentiles**	**F**	**A**	**S**	**M**	**R**	**P**	**Animals**	**Fruits**
**61-65**	5	.80	2.80	.80	1.60	.80	2.80	4.40	4.60
**Females**	10	1.60	3.60	1.00	2.00	1.00	3.00	5.60	6.00
	15	2.00	4.00	2.00	2.00	2.00	4.00	7.00	7.00
	25	4.00	6.00	4.00	4.00	4.00	6.00	9.00	9.00
	50	6.00	8.00	6.00	6.00	6.00	8.00	12.00	12.00
	75	9.00	11.00	9.00	9.00	9.00	11.00	17.00	18.00
	85	10.00	12.00	10.00	10.00	10.00	12.00	19.00	20.00
	90	11.20	13.20	11.20	11.60	11.60	13.20	20.40	20.80
	95	13.80	15.80	14.60	14.40	14.60	16.60	23.00	22.00

Supplementary Table 16. Percentiles Box by Age Range (between 61 and 65 years old), and by Educational level (Between 1 – 6 years) for females.

**Age Range (years)**	**Percentiles**	**F**	**A**	**S**	**M**	**R**	**P**	**Animals**	**Fruits**
**61-65**	5	1.00	3.00	1.00	2.00	2.00	3.00	1.00	1.00
**Males**	10	1.00	3.00	1.50	2.00	2.00	3.50	1.50	2.00
	15	1.75	3.75	2.50	2.50	2.50	4.50	2.75	4.00
	25	4.00	6.00	4.00	4.00	4.00	6.00	8.00	8.50
	50	5.00	7.00	5.50	5.50	5.00	7.50	11.50	11.00
	75	10.00	12.00	10.00	10.00	9.25	12.00	15.75	15.50
	85	12.25	14.25	11.50	11.50	12.25	13.50	20.25	20.00

	90	14.50	16.50	14.50	14.00	14.50	16.50	21.50	21.00
	95	.	.	.	.	.	.	.	.

Supplementary Table 17. Percentiles Box by Age Range (between 61 and 65 years old), and by Educational level (Between 1 – 6 years) for males.

Age Range (years)	Percentiles	F	A	S	M	R	P	Animals	Fruits
**66-70**	5	.00	2.00	.00	.00	-.80	2.00	1.20	1.40
**Female**	10	.40	2.40	.40	.40	.40	2.40	3.00	3.40
	15	1.00	3.00	1.00	1.00	1.00	3.00	5.60	5.00
	25	1.00	3.00	2.00	2.00	1.00	4.00	7.00	7.00
	50	4.00	6.00	4.00	4.00	4.00	6.00	11.00	11.00
	75	7.00	9.00	7.00	7.00	7.00	9.00	17.00	17.00
	85	9.00	11.00	9.00	10.00	10.00	11.00	19.40	19.00
	90	9.60	11.60	10.00	10.00	10.00	12.00	20.60	20.60
	95	12.80	14.80	12.80	13.80	12.80	14.80	22.00	22.00

Supplementary Table 18. Percentiles Box by Age Range (between 66 and 70 years old), and by Educational level (Between 1 – 6 years) for females.

Age Range (years)	Percentiles	F	A	S	M	R	P	Animals	Fruits
**66-70**	5	1.00	3.00	1.00	1.00	1.00	3.00	1.40	1.40
**Males**	10	1.00	3.00	1.00	1.40	1.00	3.00	3.80	3.80
	15	1.60	3.60	1.60	2.00	1.00	3.60	5.60	5.60
	25	4.00	6.00	4.00	4.00	4.00	6.00	9.00	9.00
	50	4.00	6.00	4.00	4.00	4.00	6.00	9.00	9.00
	75	6.00	8.00	6.00	7.00	6.00	8.00	14.00	14.00
	85	7.00	9.00	7.00	7.00	7.00	9.00	16.20	15.60
	90	8.20	10.20	8.20	8.20	8.20	10.20	19.80	20.40
	95	9.80	11.80	9.80	9.80	9.80	11.80	21.80	22.00

Supplementary Table 19. Percentiles Box by Age Range (between 66 and 70 years old), and by Educational level (Between 1 – 6 years) for males.

Age Range (years)	Percentiles	F	A	S	M	R	P	Animals	Fruits
**71-75**	5	1.00	3.00	1.00	2.00	1.00	3.00	6.00	5.00
**Females**	10	1.00	3.00	1.50	2.00	1.00	3.50	6.50	5.00
	15	1.00	3.00	2.00	2.00	1.25	4.00	7.00	5.25
	25	3.25	5.25	3.50	3.50	3.50	5.50	8.50	8.25
	50	4.00	6.00	4.00	4.00	4.00	6.00	10.50	10.00
	75	9.00	11.00	9.25	9.25	9.00	11.25	14.25	14.25
	85	9.00	11.00	10.00	10.00	9.75	12.00	19.50	19.50

	90	9.50	11.50	10.00	10.00	10.00	12.00	21.50	22.00
	95	.	.	.	.	.	.	.	.

Supplementary Table 20. Percentiles Box by Age Range (between 70 and 75 years old), and by Educational level (Between 1 – 6 years) for females.

Age Range (years)	Percentiles	F	A	S	M	R	P	Animals	Fruits
**71-75**	5	1.00	3.00	2.00	1.00	2.00	4.00	5.00	5.00
**Males**	10	2.50	4.50	3.00	2.50	3.00	5.00	5.00	5.50
	15	4.00	6.00	4.00	4.00	4.00	6.00	5.00	6.00
	25	4.00	6.00	4.00	4.00	4.00	6.00	5.00	6.75
	50	4.00	6.00	4.00	4.00	4.00	6.00	10.00	10.00
	75	10.00	12.00	9.00	9.25	9.25	11.00	16.00	15.50
	85	10.00	12.00	9.75	10.00	10.00	11.75	21.25	20.75
	90	12.00	14.00	12.00	11.00	11.50	14.00	22.50	22.50
	95	.	.	.	.	.	.	.	.

Supplementary Table 21. Percentiles Box by Age Range (between 70 and 75 years old), and by Educational level (Between 1 – 6 years) for males.

Age Range (years)	Percentiles	F	A	S	M	R	P	Animals	Fruits
**76-80**	5	-.90	1.10	.05	-.90	.05	2.05	3.15	3.10
**Females**	10	1.00	3.00	1.00	1.10	1.10	3.00	6.10	5.00
	15	1.45	3.45	1.45	2.30	2.30	3.45	7.00	5.15
	25	4.00	6.00	4.00	4.00	4.00	6.00	9.00	9.00
	50	6.00	8.00	6.00	6.00	6.00	8.00	12.00	12.00
	75	8.50	10.50	8.50	8.50	8.50	10.50	15.00	15.00
	85	9.00	11.00	10.00	9.85	10.00	12.00	18.40	18.40
	90	9.90	11.90	10.00	10.00	10.00	12.00	20.80	20.80
	95	13.80	15.80	13.80	12.85	11.90	15.80	21.95	21.95

Supplementary Table 22. Percentiles Box by Age Range (between 76 and 80 years old), and by Educational level (Between 1 – 6 years) for females.

Age Range (years)	Percentiles	F	A	S	M	R	P	Animals	Fruits
**76-80**	5	-	1.00	-	-1.00	.00	1.00	1.00	1.00
**Males**		1.00		1.00					
	10	-.30	1.70	-	-1.00	.00	1.00	1.70	1.00
				1.00					
	15	.55	2.55	.10	.10	.55	2.10	2.00	2.10
	25	1.00	3.00	2.00	2.00	2.00	4.00	3.25	3.25

	50	6.00	8.00	6.00	6.50	6.00	8.00	13.00	13.00
	75	9.00	11.00	9.00	9.00	9.00	11.00	16.50	16.50
	85	9.00	11.00	9.00	10.00	10.00	11.00	19.35	20.45
	90	9.30	11.30	9.30	10.00	10.00	11.30	21.30	21.00
	95	.	.	.	.	.	.	.	.

Supplementary Table 23. Percentiles Box by Age Range (between 76 and 80 years old), and by Educational level (Between 1 – 6 years) for males.

Age Range (years)	Percentiles	F	A	S	M	R	P	Animals	Fruits
**81-85**	5	1.00	3.00	1.00	2.00	1.00	3.00	2.00	3.00
**Females**	10	1.00	3.00	1.50	2.00	1.50	3.50	3.50	4.00
	15	1.75	3.75	2.50	2.50	2.50	4.50	5.50	5.00
	25	4.00	6.00	4.00	4.00	4.00	6.00	7.00	6.50
	50	5.00	7.00	5.00	5.00	5.00	7.00	10.00	10.00
	75	9.25	11.25	10.00	9.25	10.00	12.00	15.50	14.75
	85	12.25	14.25	12.25	11.50	13.00	14.25	18.50	18.50
	90	13.00	15.00	13.00	12.50	14.00	15.00	20.00	21.00
	95	.	.	.	.	.	.	.	.

Supplementary Table 24. Percentiles Box by Age Range (between 81 and 85 years old), and by Educational level (Between 1 – 6 years) for females.

Age Range (years)	Percentiles	F	A	S	M	R	P	Animals	Fruits
**81-85**	5	1.00	3.00	1.00	1.00	1.00	3.00	1.00	2.00
**Males**	10	1.70	3.70	1.00	1.00	1.00	3.00	3.80	4.10
	15	2.00	4.00	1.00	1.00	1.00	3.00	6.10	5.55
	25	2.50	4.50	2.50	2.50	1.75	4.50	9.00	9.00
	50	5.00	7.00	5.50	5.00	5.00	7.50	11.00	11.00
	75	9.00	11.00	9.00	9.00	9.00	11.00	14.25	13.50
	85	10.00	12.00	9.45	10.00	9.00	11.45	19.90	19.35
	90	11.20	13.20	10.60	10.60	9.90	12.60	21.30	21.30
	95	.	.	.	.	.	.	.	.

Supplementary Table 25. Percentiles Box by Age Range (between 81 and 85 years old), and by Educational level (Between 1 – 6 years) for males.

Age Range (years)	Percentiles	F	A	S	M	R	P	Animals	Fruits
**86-90**	5	.00	2.00	.00	-1.00	.00	2.00	5.00	6.00
**Females**									
	10	.00	2.00	.00	-1.00	.00	2.00	5.00	6.00

	15	.00	2.00	.00	-1.00	.00	2.00	5.00	6.00
	25	.25	2.25	.50	-.25	.25	2.50	6.00	6.75
	50	1.50	3.50	2.00	2.00	1.50	4.00	10.00	10.50
	75	5.75	7.75	5.00	5.75	5.75	7.00	11.75	12.00
	85	.	.	.	.	.	.	.	.
	90	.	.	.	.	.	.	.	.
	95	.	.	.	.	.	.	.	.

Supplementary Table 26. Percentiles Box by Age Range (between 86 and 90 years old), and by Educational level (Between 1 – 6 years) for females.

Age Range (years)	Percentiles	F	A	S	M	R	P	Animals	Fruits
**86-90**	5	6.00	8.00	7.00	7.00	7.00	9.00	9.00	9.00
**Males**	10	6.00	8.00	7.00	7.00	7.00	9.00	9.00	9.00
	15	6.00	8.00	7.00	7.00	7.00	9.00	9.00	9.00
	25	6.00	8.00	7.00	7.00	7.00	9.00	9.00	9.00
	50	9.00	11.00	9.00	9.00	10.00	11.00	17.00	18.00
	75	.	.	.	.	.	.	.	.
	85	.	.	.	.	.	.	.	.
	90	.	.	.	.	.	.	.	.
	95	.	.	.	.	.	.	.	.

Supplementary Table 27. Percentiles Box by Age Range (between 86 and 90 years old), and by Educational level (Between 1 – 6 years) for males.

Age Range (years)	Percentiles	F	A	S	M	R	P	Animals	Fruits
**91-95**	5	4.00	6.00	4.00	4.00	4.00	6.00	9.00	9.00
**Males**	10	4.00	6.00	4.00	4.00	4.00	6.00	9.00	9.00
	15	4.00	6.00	4.00	4.00	4.00	6.00	9.00	9.00
	25	4.00	6.00	4.00	4.00	4.00	6.00	9.00	9.00
	50	4.00	6.00	4.00	4.00	4.00	6.00	11.50	11.50
	75	4.00	6.00	4.00	4.00	4.00	6.00	.	.
	85	4.00	6.00	4.00	4.00	4.00	6.00	.	.
	90	4.00	6.00	4.00	4.00	4.00	6.00	.	.
	95	4.00	6.00	4.00	4.00	4.00	6.00	.	.

Supplementary Table 28. Percentiles Box by Age Range (between 91 and 95 years old), and by Educational level (Between 1 – 6 years) for males.

Age Range	Percentiles	F	A	S	M	R	P	Animals	Fruits

(years)									
**12-14**	5	5.45	6.45	3.45	5.45	5.00	7.00	10.45	9.45
**Females**	10	6.00	7.00	4.90	6.90	6.90	7.00	13.00	10.00
	15	7.00	7.35	6.35	7.00	7.00	8.35	13.35	11.00
	25	7.25	8.25	8.00	9.00	8.00	10.00	14.00	12.00
	50	10.00	11.00	10.00	10.00	10.00	12.00	18.00	13.00
	75	13.00	13.00	12.75	12.75	12.00	14.75	21.00	15.75
	85	14.00	15.00	14.65	14.00	14.65	15.65	22.65	16.00
	90	14.10	16.00	15.10	16.00	15.10	16.10	24.00	17.00
	95	16.00	16.00	17.00	17.55	17.00	17.55	24.00	20.00

Supplementary Table 29. Percentiles Box by Age Range (between 12 and 14 years old), and by Educational level (Between 7 – 11 years) for females.

Age Range (years)	Percentiles	F	A	S	M	R	P	Animals	Fruits
**12-14**	5	4.10	5.55	4.00	4.00	3.10	6.00	9.10	8.55
**Males**	10	6.00	6.00	5.10	5.10	5.00	6.00	12.10	10.00
	15	6.00	6.00	6.00	6.65	5.65	7.65	13.65	10.00
	25	7.00	7.00	7.00	7.75	7.00	8.75	15.00	11.00
	50	9.00	10.00	9.00	10.00	10.00	12.00	18.00	13.00
	75	12.00	14.00	12.00	14.00	14.00	16.00	22.00	16.00
	85	15.35	16.00	14.00	15.35	16.00	18.00	22.35	17.00
	90	17.80	16.00	15.00	16.90	16.90	18.90	23.00	17.90
	95	18.45	18.90	17.45	19.45	17.45	20.00	24.00	18.45

Supplementary Table 30. Percentiles Box by Age Range (between 12 and 14 years old), and by Educational level (Between 7 – 11 years) for males.

Age Range (years)	Percentiles	F	A	S	M	R	P	Animals	Fruits
**15-17**	5	4.00	6.00	5.00	6.00	5.00	6.00	10.85	8.00
**Females**	10	6.00	7.00	7.00	7.00	6.00	8.00	12.00	10.00
	15	7.00	8.00	8.00	8.55	8.00	9.00	13.00	11.00
	25	8.00	9.00	9.00	10.00	9.00	10.00	15.00	12.00
	50	11.00	11.00	11.00	12.00	11.00	13.00	18.00	13.00
	75	13.00	13.00	13.00	14.00	13.00	15.00	20.00	15.00
	85	14.00	15.00	14.00	15.00	14.00	16.00	22.00	16.00
	90	15.00	15.00	15.00	16.00	15.00	17.00	23.00	17.00
	95	16.00	16.00	15.15	18.00	17.00	19.00	24.00	18.00

Supplementary Table 31. Percentiles Box by Age Range (between 15 and 17 years old), and

by Educational level (Between 7 – 11 years) for females.

Age Range (years)	Percentiles	F	A	S	M	R	P	Animals	Fruits
**15-17**	5	4.00	5.00	4.70	5.00	5.70	6.00	9.70	7.00
**Males**	10	6.00	6.00	6.00	6.00	7.00	7.00	12.00	9.00
	15	6.10	7.00	7.00	7.10	8.00	8.10	13.00	9.00
	25	8.00	8.00	8.00	9.00	9.00	9.50	14.00	11.00
	50	10.00	11.00	10.00	11.00	11.00	12.00	17.00	12.00
	75	12.00	13.00	13.00	13.50	13.50	15.00	20.00	15.00
	85	13.00	14.90	14.00	15.00	15.00	16.00	22.00	16.00
	90	15.00	16.00	15.00	16.00	15.00	17.00	24.00	17.00
	95	18.00	17.30	16.00	17.00	16.30	20.00	24.60	19.00

Supplementary Table 32. Percentiles Box by Age Range (between 15 and 17 years old), and by Educational level (Between 7 – 11 years) for males.

Age Range (years)	Percentiles	F	A	S	M	R	P	Animals	Fruits
**18-20**	5	4.00	4.00	4.00	4.20	4.20	6.00	10.00	8.20
**Females**	10	6.00	5.00	5.00	7.00	6.00	7.00	12.00	10.00
	15	6.00	6.00	6.00	7.00	7.00	8.00	13.00	11.00
	25	7.00	8.00	8.00	9.00	8.00	10.00	14.00	12.00
	50	10.00	10.00	10.00	11.00	11.00	12.00	17.00	13.00
	75	13.00	13.00	12.00	14.00	13.00	15.00	20.00	15.00
	85	14.00	14.00	14.00	15.00	15.00	16.00	21.00	16.00
	90	15.00	15.00	15.00	16.00	15.00	17.00	22.00	17.00
	95	16.80	16.00	16.00	17.00	16.00	18.00	24.00	18.00

Supplementary Table 33. Percentiles Box by Age Range (between 18 and 20 years old), and by Educational level (Between 7 – 11 years) for females.

Age Range (years)	Percentiles	F	A	S	M	R	P	Animals	Fruits
**18-20**	5	3.00	4.00	4.00	5.00	4.00	6.00	9.00	7.00
**Males**	10	4.00	5.00	6.00	6.00	6.00	7.00	11.00	8.00
	15	5.60	6.00	6.00	7.00	6.60	8.00	12.00	9.00
	25	7.00	7.00	7.00	9.00	8.00	9.00	14.00	10.00
	50	9.00	10.00	10.00	11.00	11.00	12.00	16.00	12.00
	75	12.00	12.00	12.00	13.00	13.00	14.00	19.00	14.00
	85	13.00	13.00	13.00	14.00	14.00	16.00	21.00	15.00
	90	14.00	13.00	14.00	15.00	14.00	17.00	23.00	16.00
	95	15.00	15.00	16.00	16.00	16.00	18.00	24.00	17.00

Supplementary Table 34. Percentiles Box by Age Range (between 18 and 20 years old), and

by Educational level (Between 7 – 11 years) for males.

Age Range (years)	Percentiles	F	A	S	M	R	P	Animals	Fruits
**21-25**	5	5.00	5.00	5.00	5.00	5.00	7.00	8.50	9.00
**Females**	10	6.00	6.00	6.00	7.00	6.00	7.00	11.00	11.00
	15	7.00	7.00	7.00	8.00	7.00	9.00	12.00	11.00
	25	8.00	8.00	8.00	9.00	9.00	11.00	13.50	12.00
	50	10.00	11.00	11.00	12.00	11.00	13.00	17.00	13.00
	75	13.00	13.00	13.00	14.00	14.00	15.00	20.00	15.00
	85	14.00	15.00	15.00	16.00	15.00	17.00	23.00	16.00
	90	15.00	15.00	16.00	17.00	17.00	18.00	23.00	17.00
	95	16.00	16.00	17.00	18.00	18.00	19.50	24.00	18.00

Supplementary Table 35. Percentiles Box by Age Range (between 21 and 25 years old), and by Educational level (Between 7 – 11 years) for females.

Age Range (years)	Percentiles	F	A	S	M	R	P	Animals	Fruits
**21-25**	5	3.50	5.00	4.00	5.00	5.00	5.50	8.50	8.00
**Males**	10	5.00	6.00	6.00	6.00	6.00	7.00	11.00	9.00
	15	6.00	6.00	6.00	7.00	6.00	8.00	13.00	9.00
	25	7.00	8.00	8.00	8.00	8.50	9.00	14.00	11.00
	50	10.00	10.00	11.00	11.00	11.00	12.00	17.00	13.00
	75	13.00	13.00	13.00	14.00	13.00	14.00	20.00	15.00
	85	14.00	14.00	15.00	15.00	14.00	16.00	21.00	16.00
	90	15.00	15.00	15.00	16.00	15.00	18.00	23.00	16.00
	95	17.00	17.00	17.00	17.00	17.00	19.00	24.00	17.00

Supplementary Table 36. Percentiles Box by Age Range (between 21 and 25 years old), and by Educational level (Between 7 – 11 years) for males.

Age Range (years)	Percentiles	F	A	S	M	R	P	Animals	Fruits
**26-30**	5	4.80	2.80	4.80	5.80	3.60	5.80	11.60	7.20
**Females**	10	5.00	5.20	5.00	6.60	6.60	6.00	12.00	10.60
	15	6.00	6.00	5.00	7.00	7.00	7.40	12.40	11.00
	25	7.00	8.00	9.00	9.00	9.00	9.00	14.00	12.00
	50	11.00	11.00	11.00	11.00	12.00	12.00	15.00	14.00
	75	12.00	13.00	14.00	14.00	14.00	14.00	19.00	16.00
	85	13.60	15.60	14.60	15.00	15.00	16.60	20.00	17.00
	90	15.00	16.00	16.00	16.40	16.00	17.00	22.40	18.40
	95	16.00	16.40	18.00	17.40	18.40	19.20	24.00	20.00

Supplementary Table 37. Percentiles Box by Age Range (between 26 and 30 years old), and by Educational level (Between 7 – 11 years) for females.

Age Range (years)	Percentiles	F	A	S	M	R	P	Animals	Fruits
**26-30**	5	1.90	2.90	3.45	3.45	4.90	4.90	7.80	8.90
**Males**	10	3.90	5.80	4.90	4.00	6.00	6.00	11.80	10.00
	15	7.35	7.00	6.35	5.70	7.00	6.70	13.00	11.00
	25	8.00	8.00	8.25	9.00	8.00	9.00	14.00	12.00
	50	11.00	11.00	10.00	12.00	11.00	12.50	16.00	13.00
	75	13.50	12.00	12.75	15.00	13.00	15.75	20.00	14.00
	85	15.00	13.65	14.30	15.65	14.00	16.65	23.65	17.00
	90	17.00	16.10	16.00	17.00	15.40	18.10	24.00	18.10
	95	20.85	17.55	16.00	17.55	19.00	19.55	24.00	19.55

Supplementary Table 38. Percentiles Box by Age Range (between 26 and 30 years old), and by Educational level (Between 7 – 11 years) for males.

Age Range (years)	Percentiles	F	A	S	M	R	P	Animals	Fruits
**31-40**	5	3.00	5.00	7.00	6.00	6.00	7.00	10.00	9.00
**Females**	10	3.80	5.80	7.80	6.80	6.80	8.60	10.80	9.80
	15	6.10	6.70	8.70	7.70	7.70	9.00	11.00	10.00
	25	7.50	8.00	10.00	9.50	10.00	10.00	14.00	10.00
	50	13.00	11.00	12.00	11.00	13.00	14.00	15.00	12.00
	75	14.00	13.00	14.00	13.00	15.00	15.50	19.50	15.00
	85	15.00	14.00	14.30	14.30	15.30	16.00	20.30	16.00
	90	15.20	14.20	15.20	15.20	16.00	16.20	21.60	16.20
	95	.	.	.	.	.	.	.	.

Supplementary Table 39. Percentiles Box by Age Range (between 31 and 40 years old), and by Educational level (Between 7 – 11 years) for females.

Age Range (years)	Percentiles	F	A	S	M	R	P	Animals	Fruits
**31-40**	5	7.00	6.00	8.00	8.00	8.00	10.00	8.00	9.00
**Males**	10	7.30	6.60	8.30	8.30	8.60	10.30	8.90	9.30
	15	7.95	7.90	8.95	8.95	9.90	10.95	10.85	9.95
	25	9.00	9.25	10.25	9.25	11.00	11.00	11.75	12.00
	50	11.00	12.00	11.50	10.50	12.50	12.50	16.00	13.00
	75	14.25	13.00	14.25	15.00	14.00	15.75	18.00	16.75
	85	16.05	14.05	15.00	18.10	18.15	16.20	18.10	17.05
	90	16.70	14.70	15.00	19.40	20.10	18.80	19.40	17.70
	95	.	.	.	.	.	.	.	.

Supplementary Table 40. Percentiles Box by Age Range (between 31 and 40 years old), and by Educational level (Between 7 – 11 years) for males.

Age Range (years)	Percentiles	F	A	S	M	R	P	Animals	Fruits
**41-50**	5	1.00	3.00	2.00	2.00	1.00	4.00	5.00	5.00
**Females**	10	3.70	5.70	3.80	3.80	3.70	5.80	5.90	6.80
	15	4.00	6.00	4.00	4.00	4.00	6.00	8.55	8.70
	25	4.00	6.00	4.00	4.00	4.00	6.00	11.75	10.50
	50	7.00	9.00	6.50	7.00	6.50	8.50	16.50	16.00
	75	13.25	14.00	15.50	13.00	16.00	14.75	21.25	21.00
	85	17.00	15.60	17.15	16.15	17.30	18.15	22.00	23.00
	90	17.00	19.00	18.00	17.10	19.20	19.00	22.10	23.00
	95	.	.	.	.	.	.	.	.

Supplementary Table 41. Percentiles Box by Age Range (between 41 and 50 years old), and by Educational level (Between 7 – 11 years) for females.

Age Range (years)	Percentiles	F	A	S	M	R	P	Animals	Fruits
**41-50**	5	1.00	3.00	1.00	1.00	2.00	3.00	5.00	5.00
**Males**	10	1.00	3.00	1.00	1.00	2.00	3.00	5.00	5.00
	15	1.00	3.00	1.35	1.00	2.00	3.35	5.70	5.70
	25	1.75	3.75	2.50	1.75	2.50	4.50	7.50	7.50
	50	5.00	7.00	5.00	5.50	5.00	7.00	12.00	11.50
	75	14.50	16.50	14.25	14.50	15.25	16.25	20.25	20.25
	85	16.65	18.65	16.65	16.00	17.00	18.65	22.30	22.30
	90	.	.	.	.	.	.	.	.
	95	.	.	.	.	.	.	.	.

Supplementary Table 42. Percentiles Box by Age Range (between 41 and 50 years old), and by Educational level (Between 7 – 11 years) for males.

Age Range (years)	Percentiles	F	A	S	M	R	P	Animals	Fruits
**51-55**	5	1.00	3.00	2.00	1.00	1.00	4.00	5.65	5.00
**Females**	10	1.30	3.30	2.00	2.00	2.00	4.00	7.00	7.00
	15	4.00	6.00	4.00	4.00	4.00	6.00	9.00	9.00
	25	4.00	6.00	4.00	4.00	4.00	6.00	9.00	9.00
	50	8.00	9.50	8.00	8.00	7.00	10.00	12.00	11.50
	75	10.00	12.00	10.00	10.00	10.00	12.00	17.75	17.75
	85	12.05	14.05	13.00	13.05	13.05	15.00	20.00	20.00
	90	13.00	15.00	13.70	14.00	14.00	16.70	20.70	20.70
	95	16.00	18.00	16.35	17.00	17.00	18.35	22.00	22.00

Supplementary Table 43. Percentiles Box by Age Range (between 51 and 55 years old), and by Educational level (Between 7 – 11 years) for females.

Age Range (years)	Percentiles	F	A	S	M	R	P	Animals	Fruits
**51-55**	5	1.00	3.00	1.00	1.00	1.00	3.00	5.30	5.30
**Males**	10	1.60	3.60	1.00	1.00	1.00	3.00	6.00	6.00
	15	2.00	4.00	1.00	1.90	1.00	3.00	8.70	8.70
	25	4.00	6.00	4.00	4.00	4.00	6.00	11.00	11.50
	50	7.00	9.00	7.00	7.00	7.00	9.00	15.00	15.00
	75	10.00	12.00	9.50	10.00	10.00	11.50	18.50	18.50
	85	13.10	15.10	12.10	13.00	13.10	14.10	21.20	21.10
	90	14.80	16.80	14.60	14.20	14.80	16.60	23.00	22.40
	95	16.00	18.00	17.00	16.70	16.70	19.00	23.00	23.00

Supplementary Table 44. Percentiles Box by Age Range (between 51 and 55 years old), and by Educational level (Between 7 – 11 years) for males.

Age Range (years)	Percentiles	F	A	S	M	R	P	Animals	Fruits
**56-60**	5	1.00	3.00	1.00	1.00	1.00	3.00	4.50	4.50
**Females**	10	1.00	3.00	2.00	2.00	1.00	4.00	5.00	6.00
	15	3.00	5.00	3.00	3.00	3.00	5.00	6.00	7.00
	25	4.00	6.00	4.00	4.00	4.00	6.00	9.00	9.00
	50	9.00	11.00	9.00	9.00	9.00	11.00	15.00	15.00
	75	10.00	12.00	10.00	10.00	10.00	12.00	20.00	20.00
	85	13.00	15.00	13.00	12.50	13.50	15.00	21.00	21.00
	90	14.00	16.00	14.00	14.00	14.00	16.00	22.00	22.00
	95	16.50	18.50	16.00	16.00	16.00	18.00	22.50	23.00

Supplementary Table 45. Percentiles Box by Age Range (between 56 and 60 years old), and by Educational level (Between 7 – 11 years) for females.

Age Range (years)	Percentiles	F	A	S	M	R	P	Animals	Fruits
**56-60**	5	4.00	6.00	4.00	4.00	4.00	6.00	3.00	3.00
**Males**	10	4.00	6.00	4.00	4.00	4.00	6.00	4.20	5.40
	15	4.00	6.00	4.00	4.00	4.00	6.00	7.40	8.60
	25	6.00	8.00	6.00	6.00	6.00	8.00	12.00	11.00
	50	9.00	11.00	9.00	9.00	9.00	11.00	15.00	15.00
	75	10.00	12.00	10.00	10.00	10.00	12.00	18.00	19.00
	85	11.20	13.20	11.80	12.40	11.80	13.80	20.00	19.60

	90	14.00	16.00	14.60	15.20	14.20	16.60	21.20	20.80
	95	.	.	.	.	.	.	.	.

Supplementary Table 46. Percentiles Box by Age Range (between 56 and 60 years old), and by Educational level (Between 7 – 11 years) for males.

Age Range (years)	Percentiles	F	A	S	M	R	P	Animals	Fruits
**61-65**	5	.70	2.70	.70	.70	.70	2.70	4.10	3.80
**Females**	10	1.00	3.00	1.00	1.00	1.40	3.00	6.40	5.00
	15	1.10	3.10	1.00	1.00	2.00	3.00	7.20	7.20
	25	2.00	4.00	2.00	2.00	2.00	4.00	9.00	9.00
	50	7.00	9.00	6.00	6.00	6.00	8.00	12.00	11.00
	75	10.00	12.00	10.00	9.50	10.00	12.00	15.00	15.00
	85	10.00	12.00	10.00	10.00	10.00	12.00	18.90	20.00
	90	13.00	15.00	13.00	12.60	12.60	15.00	19.00	20.00
	95	14.60	16.60	14.20	14.60	14.90	16.20	22.00	21.30

Supplementary Table 47. Percentiles Box by Age Range (between 61 and 65 years old), and by Educational level (Between 7 – 11 years) for females.

Age Range (years)	Percentiles	F	A	S	M	R	P	Animals	Fruits
**61-65**	5	1.00	3.00	1.00	1.00	1.00	3.00	2.80	2.80
**Males**	10	1.80	3.80	1.80	1.00	1.00	3.80	4.80	4.80
	15	2.40	4.40	2.40	2.40	2.40	4.40	6.00	5.20
	25	4.00	6.00	4.00	4.00	4.00	6.00	7.00	6.00
	50	6.00	8.00	6.00	6.00	6.00	8.00	12.00	12.00
	75	9.00	11.00	9.00	10.00	9.00	11.00	17.00	17.00
	85	9.80	11.80	9.80	10.00	9.00	11.80	18.80	19.00
	90	10.00	12.00	10.00	10.00	10.00	12.00	20.20	20.40
	95	13.60	15.60	14.20	14.20	13.60	16.20	21.60	22.60

Supplementary Table 48. Percentiles Box by Age Range (between 61 and 65 years old), and by Educational level (Between 7 – 11 years) for males.

Age Range (years)	Percentiles	F	A	S	M	R	P	Animals	Fruits
**66-70**	5	1.65	3.65	2.00	1.65	1.65	4.00	3.95	4.60
**Females**	10	4.00	6.00	4.00	4.00	4.00	6.00	6.00	6.30
	15	4.00	6.00	4.00	4.00	4.00	6.00	6.95	7.00
	25	4.00	6.00	4.00	4.00	4.00	6.00	9.00	9.00
	50	6.00	8.00	6.50	6.50	7.00	8.50	14.00	14.50
	75	10.00	12.00	10.00	9.00	10.00	12.00	18.75	18.50
	85	14.00	16.00	13.05	14.00	14.00	15.05	21.05	21.05

	90	15.40	17.40	15.40	15.40	15.40	17.40	22.00	22.00
	95	16.35	18.35	16.00	16.35	16.00	18.00	23.00	23.00

Supplementary Table 49. Percentiles Box by Age Range (between 66 and 70 years old), and by Educational level (Between 7 – 11 years) for females.

Age Range (years)	Percentiles	F	A	S	M	R	P	Animals	Fruits
**66-70**	5	1.00	3.00	1.00	1.00	1.00	3.00	1.30	3.00
**Males**	10	1.60	3.60	1.60	1.00	1.60	3.60	3.80	4.20
	15	2.00	4.00	2.00	1.90	2.00	4.00	5.90	5.00
	25	4.00	6.00	4.00	4.00	4.00	6.00	8.00	8.00
	50	6.00	8.00	6.00	6.00	6.00	8.00	14.00	14.00
	75	9.00	11.00	9.50	9.00	9.50	11.50	17.50	18.50
	85	12.10	14.10	12.20	12.10	12.10	14.20	20.20	20.20
	90	13.00	15.00	14.00	13.00	13.40	16.00	22.40	22.00
	95	15.80	17.80	16.10	15.80	16.10	18.10	23.00	22.70

Supplementary Table 50. Percentiles Box by Age Range (between 66 and 70 years old), and by Educational level (Between 7 – 11 years) for males.

Age Range (years)	Percentiles	F	A	S	M	R	P	Animals	Fruits
**71-75**	5	1.00	3.00	2.00	2.00	2.00	4.00	5.00	5.00
**Females**	10	3.10	5.10	3.40	3.40	3.40	5.40	5.00	5.00
	15	4.00	6.00	4.00	4.00	4.00	6.00	5.55	5.55
	25	4.00	6.00	4.00	4.00	4.00	6.00	6.00	6.25
	50	7.00	9.00	6.00	6.50	6.00	8.00	10.00	10.00
	75	7.00	9.00	7.00	7.00	7.00	9.00	14.75	14.75
	85	9.00	11.00	10.00	9.45	10.00	12.00	17.70	18.15
	90	10.50	12.50	10.90	10.60	10.90	12.90	21.60	22.00
	95	.	.	.	.	.	.	.	.

Supplementary Table 51. Percentiles Box by Age Range (between 71 and 75 years old), and by Educational level (Between 7 – 11 years) for females.

Age Range (years)	Percentiles	F	A	S	M	R	P	Animals	Fruits
**71-75**	5	1.00	3.00	1.00	2.00	2.00	3.00	7.00	7.00
**Males**	10	1.00	3.00	1.00	2.00	2.00	3.00	7.00	7.00
	15	2.05	4.05	2.05	2.70	2.70	4.05	8.40	8.40
	25	4.00	6.00	4.00	4.00	4.00	6.00	11.25	11.00
	50	6.00	8.00	6.50	6.50	6.00	8.50	13.00	13.50
	75	7.00	9.00	7.00	7.00	6.00	9.00	17.75	18.50
	85	8.30	10.30	8.30	8.30	7.95	10.30	21.25	20.95

	90	.	.	.	.	.	.	.	.
	95	.	.	.	.	.	.	.	.

Supplementary Table 52. Percentiles Box by Age Range (between 71 and 75 years old), and by Educational level (Between 7 – 11 years) for males.

Age Range (years)	Percentiles	F	A	S	M	R	P	Animals	Fruits
**76-80**	5	1.00	3.00	1.00	2.00	1.00	3.00	6.00	6.00
**Females**	10	1.00	3.00	1.00	2.00	1.00	3.00	6.00	6.00
	15	1.05	3.05	1.05	2.00	1.00	3.05	6.25	6.25
	25	1.75	3.75	1.75	2.00	1.00	3.75	9.75	9.75
	50	4.00	6.00	4.00	4.00	4.00	6.00	11.50	11.50
	75	7.25	9.25	7.25	7.00	7.00	9.25	13.25	14.00
	85	16.35	18.35	16.35	15.40	15.40	18.35	16.75	19.60
	90	.	.	.	.	.	.	.	.
	95	.	.	.	.	.	.	.	.

Supplementary Table 53. Percentiles Box by Age Range (between 76 and 80 years old), and by Educational level (Between 7 – 11 years) for females.

Age Range (years)	Percentiles	F	A	S	M	R	P	Animals	Fruits
**76-80**	5	1.00	3.00	1.00	2.00	1.00	3.00	6.00	7.00
**Males**	10	1.00	3.00	1.00	2.00	1.00	3.00	6.00	7.00
	15	1.60	3.60	1.60	2.40	1.60	3.60	7.20	8.00
	25	4.00	6.00	4.00	4.00	4.00	6.00	12.00	12.00
	50	6.00	8.00	7.00	7.00	6.00	9.00	15.00	15.00
	75	12.00	14.00	14.00	12.00	12.00	16.00	17.00	18.00
	85	13.60	15.60	14.00	13.60	12.80	16.00	21.00	22.00
	90	.	.	.	.	.	.	.	.
	95	.	.	.	.	.	.	.	.

Supplementary Table 54. Percentiles Box by Age Range (between 76 and 80 years old), and by Educational level (Between 7 – 11 years) for males.

Age Range (years)	Percentiles	F	A	S	M	R	P	Animals	Fruits
**81-85**	5	4.00	6.00	4.00	4.00	4.00	6.00	17.00	17.00
**Females**	10	4.00	6.00	4.00	4.00	4.00	6.00	17.00	17.00
	15	4.00	6.00	4.00	4.00	4.00	6.00	17.00	17.00
	25	4.00	6.00	4.00	4.00	4.00	6.00	17.00	17.00
	50	4.00	6.00	4.00	4.00	4.00	6.00	17.00	18.50
	75	4.00	6.00	4.00	4.00	4.00	6.00	17.00	.
	85	4.00	6.00	4.00	4.00	4.00	6.00	17.00	.
	90	4.00	6.00	4.00	4.00	4.00	6.00	17.00	.

	95	4.00	6.00	4.00	4.00	4.00	6.00	17.00	.

Supplementary Table 55. Percentiles Box by Age Range (between 81 and 85 years old), and by Educational level (Between 7 – 11 years) for females.

Age Range (years)	Percentiles	F	A	S	M	R	P	Animals	Fruits
**81-85**	5	6.00	8.00	6.00	7.00	6.00	8.00	9.00	9.00
**Males**	10	6.00	8.00	6.00	7.00	6.00	8.00	9.00	9.00
	15	6.00	8.00	6.00	7.00	6.00	8.00	9.00	9.00
	25	6.00	8.00	6.00	7.00	6.00	8.00	9.00	9.00
	50	6.50	8.50	6.50	7.00	6.00	8.50	13.50	13.50
	75	.	.	.	7.00	6.00	.	.	.
	85	.	.	.	7.00	6.00	.	.	.
	90	.	.	.	7.00	6.00	.	.	.
	95	.	.	.	7.00	6.00	.	.	.

Supplementary Table 56. Percentiles Box by Age Range (between 81 and 85 years old), and by Educational level (Between 7 – 11 years) for males.

**Percentiles Box by Age Range (between 86 and 90 years old), and by Educational level (Between 7 – 11 years).**									
**Age Range (years)**	**Percentiles**	**F**	**A**	**S**	**M**	**R**	**P**	**Animals**	**Fruits**
**86-90**	5	-1.00	1.00	.00	.00	.00	2.00	6.00	5.00
**Males**	10	-1.00	1.00	.00	.00	.00	2.00	6.00	5.00
	15	-1.00	1.00	.00	.00	.00	2.00	6.00	5.00
	25	.00	2.00	.50	.50	.50	2.50	6.50	6.00
	50	1.00	3.00	1.00	2.00	1.00	3.00	7.00	7.00
	75	6.00	8.00	6.00	6.00	5.50	8.00	11.50	11.00
	85	.	.	.	.	.	.	.	.
	90	.	.	.	.	.	.	.	.
	95	.	.	.	.	.	.	.	.

Supplementary Table 57. Percentiles Box by Age Range (between 86 and 90 years old), and by Educational level (Between 7 – 11 years) for males.

Age Range (years)	Percentiles	F	A	S	M	R	P	Animals	Fruits
**41-50**	5	2.00	4.00	2.00	2.00	1.00	4.00	1.00	3.00
**Females**	10	4.00	6.00	4.00	4.00	4.00	6.00	2.00	4.00
	15	6.00	8.00	6.00	6.00	7.00	8.00	5.00	6.00
	25	6.00	8.00	6.00	6.00	7.00	8.00	9.00	9.00
	50	10.00	12.00	10.00	10.00	10.00	12.00	19.00	17.00
	75	16.00	18.00	17.00	17.00	16.00	19.00	21.00	21.00

	85	17.00	19.00	17.00	17.00	16.00	19.00	22.00	21.00
	90	17.00	19.00	17.00	17.00	17.00	19.00	23.00	22.00
	95	.	.	.	.	.	.	.	.

Supplementary Table 58. Percentiles Box by Age Range (between 41 and 50 years old), and by Educational level (<12 years) for females.

Age Range (years)	Percentiles	F	A	S	M	R	P	Animals	Fruits
**41-50**	5	4.00	6.00	4.00	4.00	4.00	6.00	1.00	1.00
**Males**	10	4.00	6.00	4.00	4.00	4.00	6.00	1.00	1.00
	15	4.15	6.15	4.15	4.15	4.15	6.15	1.85	1.95
	25	6.25	8.25	6.25	6.25	6.25	8.25	13.75	15.25
	50	16.50	18.50	16.00	16.00	16.50	18.00	20.00	21.00
	75	17.00	19.00	16.25	16.00	17.00	18.25	23.00	22.25
	85	17.00	19.00	16.95	16.00	17.00	18.95	23.00	22.95
	90	.	.	.	.	.	.	.	.
	95	.	.	.	.	.	.	.	.

Supplementary Table 59. Percentiles Box by Age Range (between 41 and 50 years old), and by Educational level (<12 years) for males.

Age Range (years)	Percentiles	F	A	S	M	R	P	Animals	Fruits
**51-55**	5	4.00	6.00	4.00	4.00	4.00	6.00	9.00	9.00
**Females**	10	6.00	8.00	6.00	6.00	6.20	8.00	9.00	9.00
	15	7.00	9.00	7.00	7.00	7.00	9.00	11.00	11.00
	25	10.00	12.00	9.50	9.50	10.00	11.50	12.00	11.50
	50	13.00	15.00	13.00	13.00	12.00	15.00	18.00	18.00
	75	16.00	18.00	16.00	16.00	16.00	18.00	21.00	21.50
	85	17.00	19.00	17.00	17.00	16.00	19.00	21.00	23.00
	90	17.00	19.00	17.00	17.00	17.00	19.00	22.00	23.00
	95	17.00	19.00	17.00	17.00	17.00	19.00	22.90	23.00

Supplementary Table 60. Percentiles Box by Age Range (between 51 and 55 years old), and by Educational level (<12 years) for females.

Age Range (years)	Percentiles	F	A	S	M	R	P	Animals	Fruits
**51-55**	5	2.40	4.40	1.60	1.60	2.40	3.60	2.60	3.40
**Males**	10	4.00	6.00	4.00	4.00	4.00	6.00	5.00	5.00
	15	5.20	7.20	5.20	5.20	5.20	7.20	5.60	5.60
	25	6.00	8.00	6.00	6.00	6.00	8.00	6.00	6.00
	50	9.00	11.00	9.00	9.00	9.00	11.00	11.00	11.00
	75	12.00	14.00	13.00	12.00	13.00	15.00	18.00	18.00
	85	12.80	14.80	13.40	14.00	13.00	15.40	20.40	21.20
	90	15.20	17.20	15.80	15.20	14.80	17.80	22.20	23.00

	95	16.00	18.00	17.00	16.00	16.00	19.00	23.00	23.00

Supplementary Table 61. Percentiles Box by Age Range (between 51 and 55 years old), and by Educational level (<12 years) for males.

Age Range (years)	Percentiles	F	A	S	M	R	P	Animals	Fruits
**56-60**	5	1.50	3.50	1.00	2.00	2.00	3.00	6.00	6.50
**Females**	10	4.00	6.00	4.00	4.00	4.00	6.00	9.00	9.00
	15	6.00	8.00	6.00	6.00	6.00	8.00	9.00	9.00
	25	6.00	8.00	6.00	6.00	7.00	8.00	10.50	10.00
	50	9.00	11.00	9.00	9.00	9.00	11.00	15.00	15.00
	75	10.00	12.00	10.00	10.00	10.00	12.00	19.50	19.50
	85	12.50	14.50	12.50	12.50	14.00	14.50	21.50	22.00
	90	14.00	16.00	14.00	14.00	14.00	16.00	22.00	23.00
	95	16.50	18.50	16.00	16.50	17.00	18.00	22.50	23.00

Supplementary Table 62. Percentiles Box by Age Range (between 56 and 60 years old), and by Educational level (<12 years) for females.

Age Range (years)	Percentiles	F	A	S	M	R	P	Animals	Fruits
**56-60**	5	1.00	3.00	1.00	1.00	2.00	3.00	5.50	6.00
**Males**	10	2.00	4.00	1.00	2.00	2.00	3.00	7.00	7.00
	15	5.00	7.00	5.00	5.00	5.50	7.00	9.00	9.00
	25	6.00	8.00	6.00	6.00	7.00	8.00	11.00	11.00
	50	12.00	14.00	12.00	12.00	12.00	14.00	20.00	19.00
	75	16.00	18.00	16.00	16.00	16.00	18.00	21.00	22.00
	85	16.00	18.00	17.00	17.00	16.50	19.00	22.00	23.00
	90	16.00	18.00	17.00	17.00	17.00	19.00	22.00	23.00
	95	17.00	19.00	17.00	17.00	17.00	19.00	23.00	23.00

Supplementary Table 63. Percentiles Box by Age Range (between 56 and 60 years old), and by Educational level (<12 years) for males.

Age Range (years)	Percentiles	F	A	S	M	R	P	Animals	Fruits
**61-65**	5	1.15	3.15	2.10	2.10	2.10	4.10	1.25	2.20
**Females**	10	4.50	6.50	4.50	4.50	4.50	6.50	6.50	6.50
	15	9.00	11.00	9.00	9.00	9.00	11.00	11.15	11.15
	25	9.25	11.25	9.00	9.00	9.00	11.00	14.25	14.00
	50	12.00	14.00	12.50	12.00	12.50	14.50	18.00	18.50
	75	16.00	18.00	16.00	16.00	16.00	18.00	21.00	22.00
	85	16.00	18.00	16.00	17.00	17.00	18.00	21.85	23.00
	90	16.90	18.90	16.90	17.00	17.00	18.90	22.90	23.00
	95	17.00	19.00	17.00	17.00	17.00	19.00	23.00	23.00

Supplementary Table 64. Percentiles Box by Age Range (between 61 and 65 years old),

and by Educational level (<12 years) for females.

**Age Range (years)**	**Percentiles**	**F**	**A**	**S**	**M**	**R**	**P**	**Animals**	**Fruits**
**61-65**	5	1.00	3.00	1.00	1.00	1.00	3.00	5.00	6.00
**Males**	10	2.50	4.50	2.50	2.50	2.50	4.50	6.00	6.50
	15	4.00	6.00	4.00	4.00	4.00	6.00	9.00	9.00
	25	4.00	6.00	4.00	4.00	4.00	6.00	17.25	16.50
	50	9.00	11.00	9.50	10.00	10.00	11.50	20.00	19.50
	75	13.75	15.75	13.75	14.75	14.00	15.75	22.25	22.25
	85	16.75	18.75	16.75	17.00	17.00	18.75	23.00	23.00
	90	17.00	19.00	17.00	17.00	17.00	19.00	23.00	23.00
	95	.	.	.	.	.	.	.	.

Supplementary Table 65. Percentiles Box by Age Range (between 61 and 65 years old), and by Educational level (<12 years) for males.

**Age Range (years)**	**Percentiles**	**F**	**A**	**S**	**M**	**R**	**P**	**Animals**	**Fruits**
**66-70**	5	4.00	6.00	4.00	4.00	4.00	6.00	7.00	5.00
**Females**	10	4.20	6.20	4.20	4.30	4.20	6.20	7.70	5.90
	15	5.30	7.30	5.30	5.95	5.30	7.30	11.55	10.85
	25	6.75	8.75	6.75	7.00	6.75	8.75	14.00	14.00
	50	11.00	13.00	10.50	10.50	11.50	12.50	15.00	15.00
	75	16.00	18.00	16.00	17.00	16.00	18.00	21.00	21.25
	85	16.35	18.35	16.00	17.00	16.35	18.00	21.35	22.35
	90	16.90	18.90	16.00	17.00	16.90	18.00	21.90	22.90
	95	.	.	.	.	.	.	.	.

Supplementary Table 66. Percentiles Box by Age Range (between 66 and 70 years old), and by Educational level (<12 years) for females.

**Age Range (years)**	**Percentiles**	**F**	**A**	**S**	**M**	**R**	**P**	**Animals**	**Fruits**
**66-70**	5	4.00	6.00	4.00	4.00	4.00	6.00	9.00	9.00
**Males**	10	4.60	6.60	4.90	4.90	4.60	6.90	9.60	9.60
	15	5.90	7.90	6.85	6.85	5.90	8.85	10.90	10.90
	25	7.50	9.50	7.50	7.75	6.75	9.50	11.75	12.50
	50	10.00	12.00	9.50	10.00	10.00	11.50	16.50	17.00
	75	13.00	15.00	13.00	13.00	12.75	15.00	21.75	21.00
	85	14.10	16.10	14.10	14.15	14.15	16.10	23.00	22.05
	90	15.40	17.40	15.40	16.10	16.10	17.40	23.00	22.70
	95	.	.	.	.	.	.	.	.

Supplementary Table 67. Percentiles Box by Age Range (between 66 and 70 years old), and by Educational level (<12 years) for males.

Age Range (years)	Percentiles	F	A	S	M	R	P	Animals	Fruits
**71-75**	5	4.00	6.00	4.00	4.00	4.00	6.00	7.00	7.00
**Females**	10	4.00	6.00	4.00	4.00	4.00	6.00	7.00	7.00
	15	4.15	6.15	4.10	4.10	4.10	6.10	7.10	7.10
	25	6.25	8.25	5.50	5.50	5.50	7.50	8.50	8.50
	50	9.50	11.50	10.00	9.50	9.50	12.00	13.00	13.50
	75	14.50	16.50	14.75	13.75	14.75	16.75	17.25	18.00
	85	15.90	17.90	16.85	15.85	16.85	18.85	17.95	18.00
	90	.	.	.	.	.	.	.	.
	95	.	.	.	.	.	.	.	.

Supplementary Table 68. Percentiles Box by Age Range (between 71 and 75 years old), and by Educational level (<12 years) for females.

Age Range (years)	Percentiles	F	A	S	M	R	P	Animals	Fruits
**71-75**	5	1.00	3.00	1.00	2.00	1.00	3.00	6.00	5.00
**Males**	10	1.00	3.00	1.00	2.00	1.00	3.00	6.00	5.00
	15	2.05	4.05	2.05	2.70	2.05	4.05	6.35	5.35
	25	4.50	6.50	4.50	4.75	4.50	6.50	7.50	6.75
	50	12.00	14.00	13.00	12.50	12.50	15.00	14.50	14.50
	75	15.25	17.25	16.25	15.50	15.50	18.25	19.25	19.25
	85	16.00	18.00	17.00	16.65	16.65	19.00	21.30	20.65
	90	.	.	.	.	.	.	.	.
	95	.	.	.	.	.	.	.	.

Supplementary Table 69. Percentiles Box by Age Range (between 71 and 75 years old), and by Educational level (<12 years) for males.
